# Quercetin Impacts Expression of Metabolism- and Obesity-Associated Genes in SGBS Adipocytes

**DOI:** 10.3390/nu8050282

**Published:** 2016-05-12

**Authors:** Andreas Leiherer, Kathrin Stoemmer, Axel Muendlein, Christoph H. Saely, Elena Kinz, Eva M. Brandtner, Peter Fraunberger, Heinz Drexel

**Affiliations:** 1 Vorarlberg Institute for Vascular Investigation and Treatment (VIVIT), Feldkirch A-6800, Austria; andreas.leiherer@vivit.at (A.L.); axel.muendlein@vivit.at (A.M.); christoph.saely@lkhf.at (C.H.S.); elena.kinz@vivit.at (E.K.); lilli.brandtner@vivit.at (E.M.B.); 2 Private University of the Principality of Liechtenstein, Triesen FL-9495, Liechtenstein; kathrin.stoemmer@gmx.de (K.S.); pfraunberger@mzl.at (P.F.); 3 Medical Central Laboratories, Feldkirch A-6800, Austria; 4 Department of Medicine and Cardiology, Academic Teaching Hospital Feldkirch, Feldkirch A-6800, Austria; 5 Drexel University College of Medicine, Philadelphia, PA 19104, USA

**Keywords:** quercetin, phytochemicals, enolase 2, ENO2, angiopoietin-like 4, ANGPTL4, plasminogen activator inhibitor-1, PAI-1, SERPINE1, phosopho-fructokinase, PFKP, 6-phosphofructo-2-kinase/fructose-2,6-biphosphatase 4, PFKFB4, complement factor D, adipsin, CFD, fibronectin type III domain-containing 5, irisin, FNDC5, interleukin-1β, IL1B

## Abstract

Obesity is characterized by the rapid expansion of visceral adipose tissue, resulting in a hypoxic environment in adipose tissue which leads to a profound change of gene expression in adipocytes. As a consequence, there is a dysregulation of metabolism and adipokine secretion in adipose tissue leading to the development of systemic inflammation and finally resulting in the onset of metabolic diseases. The flavonoid quercetin as well as other secondary plant metabolites also referred to as phytochemicals have anti-oxidant, anti-inflammatory, and anti-diabetic effects known to be protective in view of obesity-related-diseases. Nevertheless, its underlying molecular mechanism is still obscure and thus the focus of this study was to explore the influence of quercetin on human SGBS (Simpson Golabi Behmel Syndrome) adipocytes’ gene expression. We revealed for the first time that quercetin significantly changed expression of adipokine (Angptl4, adipsin, irisin and PAI-1) and glycolysis-involved (ENO2, PFKP and PFKFB4) genes, and that this effect not only antagonized but in part even overcompensated the effect mediated by hypoxia in adipocytes. Thus, these results are explained by the recently proposed hypothesis that the protective effect of quercetin is not solely due to its free radical-scavenging activity but also to a direct effect on mitochondrial processes, and they demonstrate that quercetin might have the potential to counteract the development of obesity-associated complications.

## 1. Introduction

Obesity confers a high risk of developing numerous metabolic and cardiovascular complications. In the context of extensively growing or prevailing adipose tissue, biochemical and cellular changes take place in adipocytes, in the presence of reduced oxygen supply [1]. Hypoxia is a major starting point of the inflammatory process in adipose tissue and modulates adipocyte metabolism [2,3,4,5]. Insufficient oxygen supply is sensed via the mitochondrial electron transfer chain and thus reactive oxygen species (ROS) production is extremely elevated leading to stabilization of hypoxia inducible transcription factors [6], thereby taking over the key role in the activation of signaling pathways that are relevant for further metabolic adaptation and adipokine secretion in adipocytes resulting in a dysfunction of adipose tissue [7]. There is evidence that this dysregulation of metabolism in adipose tissue under hypoxia promotes insulin resistance and dyslipidemia and consequently initiates the development of diabetes and cardiovascular disease [2,8].

For that reason, there is growing interest worldwide in plant compounds with respect to their potential to combat obesity and subsequent diseases. Among the more than 4000 flavonoids [9] quercetin is the most common one. Found in fruits, vegetables, wine, tea, and nuts, it represents a central part of our diet [10]. It is regarded as the most effective scavenger of ROS [11] as well. Molecular effects of this phytochemical are poorly understood, although the mitochondrial membrane seems to play a major role herein [10]. The present work was aimed at elucidating the effect of quercetin on the expression of adipokine, glycolytic, and inflammatory genes in hypoxic human Simpson Golabi Behmel Syndrome (SGBS) adipocytes.

## 2. Materials and Methods

### 2.1. Cell Culture and Reagents

Human SGBS preadipocytes were kindly provided by Dr. M. Wabitsch [12] and have been cultivated as described previously [12]. Briefly, cells were maintained in 15 mL DMEM/Ham’s F12 (1:1) medium (Invitrogen, Paisley, UK) containing 10% fetal calf serum (FCS; Invitrogen), 100 U/mL penicillin (Invitrogen), 100 μg/mL streptomycin (Invitrogen), 33 μM biotin, and 17 μM pantothenate. To differentiate SGBS cells into adipocytes, near confluent cells were washed three times with phosphate buffered saline (PBS) and cultured in FCS-free differentiation medium: DMEM/Ham’s F12 (1:1) medium supplemented with 100 U/mL penicillin, 100 μg/mL streptomycin, 33 μM biotin, 17 μM pantothenate, 10 μg/mL human transferrin, 10 nM insulin, 100 nM hydrocortisone, 0.2 nM triiodothyronine, 25 nM dexamethasone, 500 μM 3-isobutyl-1-methylxanthine (IBMX), and 2 μM rosiglitazone. After four days, this medium was replaced by differentiation medium excluding dexamethasone, IBMX, and rosiglitazone, which was changed every three to four days. At day 12 after induction of differentiation, 25 μM quercetin (Q4951-10G, LOT#SLBD8415V, Sigma-Aldrich, Steinheim, Germany), dissolved in 23 μL DMSO, was added to cell cultures (15 mL), and 23 μL DMSO without quercetin was added to control-cultures (15 mL), resulting in a 0.15% (*v*/*v*) DMSO concentration in all cell cultures. All cell cultures were cultivated for another 48 h and then exposed to hypoxia. To create a hypoxic environment (1% O_2_), cells were placed in a MIC-101 modular incubator chamber (Billups-Rothenberg, Inc., Del Mar, CA, USA), flushed with a mixture of 1% O_2_, 5% CO_2,_ and 94% N2, sealed, and incubated at 37 °C. Adipocytes were cultured in hypoxic environment for 16 h, whereas control groups were cultured under normoxic conditions (21% O_2_). In total we had four different treatment groups, and each of these approaches consisted of four independent experiments. Reagents were obtained from Sigma-Aldrich unless specified otherwise.

### 2.2. Cell Lysis

Total RNA was prepared from cell samples using the RNeasy Lipid Tissue kit according to the manufacturer’s instructions (Qiagen, Hilden, Germany), including the optional DNase step. RNA quantity and purity was determined on NanoDropTM 2000 (Thermo Scientific, Waltham, MA, USA).

### 2.3. Quantitative PCR

RNA was reverse transcribed using the SuperScript III First-Strand Synthesis Kit (Invitrogen) and quantitative PCR (qPCR), was performed using the PowerSYBR^®^ Green PCR Master Mix (Thermo Scientific) on LightCycler^®^ 480 System (Roche Diagnostics, Rotkreuz, Switzerland). The primers were synthesized by Microsynth (Balgach, Switzerland) (sequences are disclosed in the supplementary section). A melting curve profile was processed after each run to confirm specific transcripts. All reactions were performed in triplicates and the samples were normalized to the endogenous reference TATA-binding protein (TBP) values.

### 2.4. Data Analysis

Results were calculated as cycle threshold values relative to controls according to the ∆∆Ct method and expressed as fold change (FC, 2^−∆∆Ct^). Standard deviation of FC (SD) has been calculated according to range for target relative to calibrator resulting from incorporating the standard deviation s of the ∆∆Ct values into the fold-difference calculation: 2^−∆∆Ct^ with ∆∆Ct + s, and ∆∆Ct − s. Statistical data analysis was performed using IBM SPSS (version 22, Armonk, NY, USA). Normal distribution of data was confirmed by the Shapiro-Wilks test. To test for significant differences, we used one-way ANOVA to see if there were between-group differences. As there were significant differences in all cases, we proceeded to *post-hoc* testing by multiple comparison Bonferroni testing. *p*-values smaller than 0.05 were considered significant.

## 3. Results

**Figure 1:**
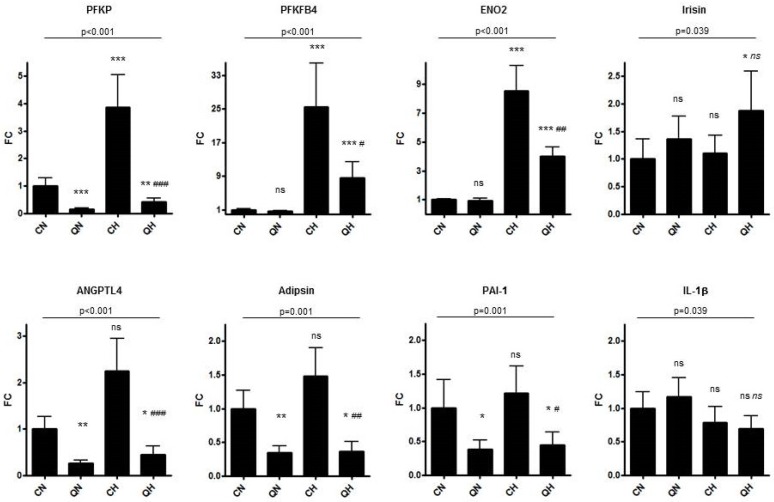
Impact of quercetin on gene transcription of normoxic and hypoxic adipocytes. Levels of mRNA were assessed in SGBS adipocytes cultivated in the presence (**Q**) or absence of quercetin (**C**) under normoxic (**N**) or hypoxic (1% O_2_) conditions (**H**). Transcriptional alterations are expressed as fold change (**FC**) with standard deviation (2^−∆∆ct^ with ∆∆ct + s and ∆∆ct − s, where s is the standard deviation of the ∆∆ct value) relative to cultivation without quercetin in a normoxic atmosphere (CN). All data represent the mean of four independent experiments, each consisting of triplicates. TATA binding protein (TBP) has been used as a reference gene. According to one-way ANOVA, there were significant differences for all gene expression sets with respect to the four treatment groups (*p*-values are indicated). For analyzing differences between two treatment groups post hoc analysis according to Bonferroni was used. A *p*-value < 0.05, is marked as *, a *p*-value < 0.01 as **, and a *p*-value < 0.001 as *** for the comparison of QN, CH, or QH to CN. For the comparison of QH to CH a *p*-value < 0.05 is marked as #, a *p*-value < 0.01 as ^##^, and a *p*-value < 0.001 as ^###^.

In the present study we focused on the expression of genes which are known or suspected to be impacted by a hypoxic environment in adipose tissue. In addition, genes were selected based on (i) their role in glucose metabolism and inflammation; (ii) their function as adipokines; and (iii) their role in obesity-associated diseases such as diabetes. These genes were enolase 2 (ENO2), angiopoietin-like 4 (ANGPTL4), plasminogen activator inhibitor-1 (PAI-1, also known as SERPINE1), platelet-type 6-phosophofructokinase (PFKP), 6-phosphofructo-2-kinase/fructose-2,6-biphosphatase 4 (PFKFB4), complement factor D (CFD, also known as adipsin), fibronectin type III domain-containing 5 (FNDC5, which encodes the precursor of irisin), and interleukin-1β (IL1B). Expression of the respective genes was assessed in differentiated, mature SGBS adipocytes, which were cultivated at 37 °C under normoxia (N) or hypoxia (H) in media supplemented with (Q) or without 25 μM quercetin (C) for 16 h. Results of gene expression analysis for the four treatment groups (CN, QN, CH, and QH) are summarized in Figure 1 and the supplementary section.

For each gene, mRNA levels were assessed to investigate the effect of quercetin under normoxic cultivation (QN) compared to normoxic cultivation without quercetin (CN), the effect of hypoxic cultivation without quercetin (CH) compared to normoxic cultivation without quercetin (CN), the effect of both quercetin supplementation and hypoxic cultivation (QH) compared to normoxic cultivation without quercetin (CN), and the effect of quercetin under hypoxic cultivation (QH) compared to hypoxic cultivation without quercetin (CH, see supplementary section).

Cultivation of the SGBS adipocytes under hypoxia significantly increased expression of ENO2, PFKP, and PFKFB4 compared to cultivation under normoxia. No significant effect of hypoxia was seen for gene expression of FNDC5/irisin, of adipokines PAI-1 and CFD/adipsin, nor of IL-1β (see supplementary data), though expression of ANGPTL4 was just failing significance (*p* = 0.057). In contrast, after the supplementation of normoxic cultivated culture samples with 25 μM quercetin, we observed a significant decrease in the expression of ANGPTL4, CFD/adipsin, PAI-1, and PFKP, compared to normoxic cultivation without quercetin. In samples cultivated under hypoxia and supplemented with quercetin, compared to samples cultivated under hypoxia but without quercetin, we observed a significant decrease in ANGPTL4, CFD/adipsin, PAI-1, and PFKP, and additionally in PFKFB4 and ENO2. The strongest impact of quercetin supplementation, both under normoxic as well as hypoxic conditions, was observed for PFKP, as indicated by a 6.5 fold decrease of gene expression under normoxia (FC = 0.155; *p* = 6.9 × 10^−6^) and a 9.2 fold decrease under hypoxia (FC = 0.109, *p* = 2.6 × 10^−6^). When comparing gene expression of quercetin-treated and hypoxic cultivated samples to samples cultivated under normoxia and without quercetin, we still found a significant inhibition of ANGPTL4, CFD/adipsin, PAI-1, and PFKP, whereas FNDC5/irisin, ENO2, and PFKFB4 gene expression was significantly raised instead.

## 4. Discussion

This work examined the regulatory impact of quercetin on the gene expression of human SGBS adipocytes. We demonstrated that quercetin is able to significantly decrease gene expression of adipokines ANGPTL4, adipsin, and PAI-1 as well as of glycolysis-associated enzymes ENO2, PFKP, and PFKFB4. Each of these is assumed to be involved in the development of obesity-associated complications.

The most striking effect was observed on the platelet-type 6-phosphofructokinase gene PFKP. It is involved in glycolysis, catalyzing fructose 6-phosphate to fructose1,6-bisphophate conversion. Elevated PFKP expression is known to be associated with increased body mass index (BMI) and obesity [13,14]. PFKP enzyme activity is inhibited by ATP, citrate, fatty acids [15], and by new synthetic molecules presently undergoing clinical trials [16,17]. We could clearly demonstrate that its expression is upregulated by hypoxia, which is, most likely, due to a direct binding of HIF-1α [3] and downregulated by quercetin, whereby the latter effect was predominant when both factors were applied in parallel.

The enolase 2 gene ENO2 is directly involved in glycolysis, catalyzing the reversible conversion of 2-phosphoglycerate to phosphoenolpyruvate. Similar to PFKP, ENO2 gene expression was significantly decreased by quercetin treatment under hypoxic conditions, but in contrast to PFKP, the attenuation by quercetin could not outperform the hypoxia-effect. The same applies to the expression of 6-phosphofructo-2-kinase/fructose-2,6-biphosphatase gene PFKFB4. It regulates the steady-state concentration of 2,6-bisphosphate, an allosteric activator of phosphofructokinase. Like PFKP and ENO2, it is activated by hypoxia as well [3,18,19,20]. In metabolic screens PFKFB4 has been proposed as a new potential target in cancer therapy as its silencing increased the ROS level and inhibited survival of cancer cells but not epithelial cells. Thus PFKFB4 seems to be essential to keep balance between glycolytic activity and antioxidant production at least in cancer cells [21]. Whether hypoxia-mediated upregulation of PFKFB4 might prevent ROS overproduction in adipocytes is unknown, but appears contradictory.

The fasting induced adipose factor ANGPTL4 is predominantly produced in adipose tissue. It is a target of peroxisome proliferator-activated receptor (PPAR) γ [22] and recently it has been demonstrated to be inhibited by AMP-activated kinase (AMPK) activation [23,24]. It is an important player in energy metabolism and insulin sensitivity and its overexpression elevates triglycerides and total cholesterol and impacts the activity of mitochondrial respiratory chain complexes [25]. Although its role in the context of metabolic diseases is still elusive, ANGPTL4 plasma levels have been reported to be significantly higher in patients with metabolic syndrome and were predictive for future cardiovascular events [26]. In the present study, our data demonstrated a trend for hypoxia-mediated upregulation. Quercetin treatment, in contrast, led to a significant decrease of ANGPTL4 expression and that inhibiting effect was not abolished even in the presence of hypoxia.

Similarly, PAI-1 is also a target of PPARγ [27,28]. Quercetin has previously been described to activate AMPK and to decrease PPARγ expression [29]. AMPK activation is known to result in PPARγ inhibition [30,31,32]. As quercetin also exhibits the ability to decrease ATP production, causing an increase in AMP and the activation of the AMPK signaling pathway [33], a common regulatory mechanism for ANGPTL4 and PAI-1 appears evident. It is presently known that circulating PAI-1 levels are increased in the metabolic syndrome as well, and that they are strongly associated with visceral adiposity and may contribute to the inflammatory state in obesity [34]. Moreover, mice lacking PAI-1 have increased energy expenditure, improved glucose tolerance, enhanced insulin sensitivity, and are resistant to diet- or genetically induced obesity. Improvement of insulin sensitivity by weight loss or treatment with insulin sensitizers such as metformin or thiazoladinediones significantly reduces circulating PAI-1 levels [34]. Thus PAI-1 has been considered as a biomarker to predict obesity-associated diseases [35]. Apart from the role as a marker, the PAI-1-PPARγ interaction may also be a potential target for novel anti-obesity drugs. In that context, our findings, which indicate that there is a significant downregulation of PAI-1 expression upon quercetin treatment independent of normoxic or hypoxic cultivation, together with the previously reported PAI-1 downmodulation by resveratrol [36], may be helpful in elucidating the detailed mode of action of these phytochemicals for future clinical use.

Knowledge about the adipokine adipsin, which is encoded by the CFD gene, is very limited. It is the rate-limiting enzyme of the alternative complement pathway, working as serine protease [37], and it is mainly produced by adipocytes [38]. It is known that adipsin levels are associated with BMI, but the way its expression is regulated is presently unknown [39]. In the context of macular degeneration, a decrease of adipsin [40] and an inhibition of the systemic activation of the complement system [41] have both been observed in the plasma of affected patients upon treatment with the anti-oxidant lutein. Whether that anti-oxidant affects gene expression of CFD/adipsin in adipocytes is not known [39]. Here, we are able to describe for the first time a downmodulation of CFD/adipsin by the anti-oxidant quercetin in adipocytes. Thus these results are of broad clinical interest.

FNDC5, primarily identified as a myokine which is cleaved and secreted as irisin from muscle during exercise is known to induce metabolic benefits after exercise [42]. A recent study reported that white adipose tissue in humans and rats is able to express and secrete FNDC5/irisin as well [43]. In line with data from a human trial reporting no effect of hypoxia on irisin levels [44], we also did not observe a significant effect of hypoxic treatment on the expression of FNDC5/irisin in our study. Interestingly, we observed a significant increase of FNDC5/irisin expression by quercetin supplementation under hypoxia and there is, to our knowledge, no other study reporting the impact of phytochemicals on FNDC5/irisin in adipocytes. Of interest, FNDC5 expression is, in contrast to ANGPTL4 and PAI-1, known to be elevated by AMPK activation [42]. In line with our data, a similar effect on FNDC5/irisin expression was seen in the hippocampus tissue of rats receiving quercetin leading to a protection against brain damage under hypoxia [45]. Thus these findings may together initiate future studies further elucidating the overall function of irisin in cell types other than myocytes.

We also observed that neither quercetin nor hypoxic cultivation did impact the expression of IL-1β. This is, in case of the latter, a bit surprising as hypoxia in adipose tissue is suggested to induce inflammation. However, similar results have been previously reported in human adipocytes [4,46]. Drawing conclusions from adipocytes about adipose tissue is problematic and does not take into account the complex *in vivo* situation with various cell-type interactions involved in triggering and regulating the inflammatory cascade. That issue has been reviewed in detail recently [47].

Quercetin has a radical scavenging capacity [48] and its pure stoichiometric consumption of free radicals by the molecule structure itself has the potential to antagonize hypoxia [49]. Hypoxia is sensed by mitochondria and leads to an increase in ROS generation [6]. ROS overproduction has previously been hypothesized to trigger a couple of independent pathways implicated in metabolism [50]. Such chronic oxidative stress plays a pivotal role in the pathogenesis of degenerative disorders [51]. On a molecular level, the rise of ROS levels leads to the stabilization of HIF-1 [6,52]. Hence the antioxidant capacity of quercetin might be responsible for a decreased expression of HIF-1-dependent genes like PFKFB4 [19], PFKP, and ENO2 [3]. This property of quercetin is in line with the reduced expression of PFKFB4 and ENO2 in our study and resembles the effect of a HIF-inhibitor [3]. However, it does not explain why gene expression in the case of PFKP or ANGPTL4 drops below the control level, outperforming by far the opposed effect of hypoxia. Likewise, the expression of CFD/adipsin and PAI-1 was significantly decreased as well, although hypoxia had only a slight and insignificant effect.

In order to tie all results together, we believe that quercetin action, which not only antagonizes but also outperforms the effect mediated by hypoxia, lies not only in its radical scavenging capacity, but is mainly based on intracellular mechanisms [33]. Quercetin is known to accumulate in mitochondria [53], it has previously been hypothesized by us [10] and has recently become more commonly accepted that quercetin influences the mitochondrial electron transfer chain [33,54]. In addition, this seems to be associated with quercetin’s action on mitochondrial biogenesis and apoptosis but also on the mitochondrial permeability transition pore, the membrane potential, and finally ATP generation impacting the AMPK activity [29,45,55,56,57,58,59,60,61,62,63]. Of interest, Lago *et al.* have revealed complex I as a target of structural binding by quercetin competing with coenzyme Q [64]. Such a specific effect on the mitochondria may be associated with or causative for further downstream effects on different targets including AMPK activation. Thus the effects of quercetin as seen in our study may be related to its (i) radical scavenging activity which would appear to counteract hypoxia, but also to its (ii) direct binding of the mitochondrial electron transfer chain complexes, probably impacting mitochondrial function including energy homeostasis, which finally leads to the mentioned effects on AMPK-dependent targets.

Nevertheless, we have to mention that the detailed role of quercetin in hypoxic adipose tissue is still elusive and there are several mechanism including the inhibition of the proteasome [65], the modulation of the JNK and ERK pathway as well as AP-1 and NF-κB activation, and the ambiguous role of PPARγ [29,66] which need further investigation. Moreover, since the potential of human adipose tissue to differentiate is limited, SGBS adipocytes were used. They are a valuable, well established and relevant tool to study human adipocyte biology *in vitro* [12,67,68,69,70,71,72,73]. SGBS adipocytes are derived from the stromal cells fraction of subcutaneous adipose tissue of a patient suffering from Simpson-Golabi-Behmel syndrome, and feature a long lasting and high capacity for adipose differentiation and at the same time display a gene expression pattern similar to mature fat cells [12,74]. They have been used to explore the effect of hypoxia on adipose tissue [3,68,75,76,77,78] and a comprehensive gene expression and secretome profiling under hypoxic conditions has already been done [4,79]. However we cannot exclude that the underlying mutation in the SGBS cells coming from the specimen of a diseased patient [67] may impact gene expression in a different way than it would be seen in adipocytes from a healthy subject. Therefore, additional studies using, for example, primary cells derived from lipoaspirates are needed to be performed to further elucidate the relation of whole adipose tissue hypoxia and the chronic inflammation observed in obesity.

Finally, the amount of quercetin used to treat SGBS adipocytes in the present study was 25 μM and that concentration was comparable to those from previous *in vitro* studies [10]. The estimated quercetin intake by Western diet ranges from 0 to 30 mg [80], whereas up to 2000 mg have been administered in clinical trials [10]. However, it has to be mentioned that quercetin bioavailability is low and varies widely between individuals due to endogenous and exogenous factors [81]. The utility of nanoparticles as delivery carriers for quercetin has been recently summarized by Nam *et al.* (2016) [82]. Such a nanoformulation demonstrates the ability to enhance solubility of quercetin in water, absorption into the body, circulation time, and target specificity. Thus these more stable and long-lived application forms may further release and potentiate quercetin’s putative health benefits [82].

In conclusion, this study demonstrated that quercetin is able to antagonize and, in part, to overcome effects mediated by hypoxia. These results are in accordance with the hypothesis recently proposed by de Oliveira *et al.* suggesting that a direct free radical-scavenging activity of quercetin cannot be concluded as the major mechanism for the clinical effects of quercetin, and that there must be a direct effect in mitochondrial processes [33]. In addition, they further substantiate and elucidate quercetin’s anti-diabetic effect and suggest that quercetin may play a protective role counteracting the development of obesity-associated associations.

## Supplementary Materials

The following are available online at http://www.mdpi.com/2072-6643/8/5/282/s1, Table S1: Oligonucleotide sequences. All sequences are given in 5′-3′ orientation, Table S2: Gene expression data according to qPCR analysis.
