# Supplementary material for: Quercetin Impacts Expression of Metabolism- and Obesity-Associated Genes in SGBS Adipocytes
Source: Nutrients. 2016 May 12;8(5):282. doi: 10.3390/nu8050282 (PMC4882695; doi:10.3390/nu8050282)
Supplement: Supplementary file 1 [file nutrients-08-00282-s001.docx]

Supplementary Materials: Quercetin Impacts Expression of Metabolism- and Obesity-Associated Genes in SGBS Adipocytes

Andreas Leiherer, Kathrin Stoemmer, Axel Muendlein, Christoph H. Saely, Elena Kinz, Eva M. Brandtner, Peter Fraunberger and Heinz Drexel

**Table S1.** Oligonucleotide sequences. All sequences are given in 5′-3′ orientation.

| **Oligonucleotides** **Gene** | **Sequence** |
| --- | --- |
| ANGPTL4 | fwd ATGGCTCAGTGGACTTCAACC |
|  | rev ATGCTATGCACCTTCTCCAGAC |
| CFD | fwd ACGGGAAGGTGCAGGTTCT |
|  | rev TGTAGCAGCAGGAGGTCGTG |
| PAI-1 | fwd CAGAAAGTGAAGATCGAGGTGAA |
|  | rev CGGACCACAAAGAGGAAGG |
| IL-1β | fwd TGAAAGATGATAAGCCCACTCTACA |
|  | rev AGACTCAAATTCCAGCTTGTTATTG |
| ENO 2 | fwd CATGTGGCTGTAGATCCCAAG |
|  | rev ACGCAGGCTTCAGTGAGTACAC |
| PFKP | fwd CGATGATTCCATTTGTGTGC |
|  | rev AGCTTGAGCCACCACTGTTC |
| PFKFB4 | fwd CTCCTGTGGCATATGGTTG |
|  | rev AGGTCTTGAGATGTCCACG |
| FNDC5 | fwd AGGTAACCATGAAAGAGATGGG |
|  | rev CTTGATGATGTCATACTGGCG |

**Table S2.** Gene expression data according to qPCR analysis. Cell cultures were supplemented with 25 μM Quercetin in DMSO (**Q**) or with DMSO alone as control (**C**) under normoxic (**N**) or hypoxic conditions (**H**). Quantitative PCR (qPCR) was performed analyzing mRNA levels of mentioned genes with TBP used as reference gene. ∆ct is given as the mean of four independent experiments, each consisting of triplicates. Gene expression, given as ∆∆ct or FC (fold change) respectively, has been determined using the normoxic treatment group without quercetin (CN, calibrator1), or the hypoxic treatment group without quercetin (CH, calibrator2). FC range (2^−∆∆ct^ with ∆∆ct + s and ∆∆ct − s, where s is the standard deviation of the ∆∆ct value) is represented by ±SD. ANOVA was used to detect overall significant differences, and respective p-values are given (*). For comparing between different treatment groups, ANOVA with post hoc testing (Bonferroni) was applied and the respective p-values result from correction for multiple comparison (**). ANGPTL4 denotes Angiopoietin-like like 4, CFD the complement factor D (Adipsin), FNDC5 the irisin precursor fibronectin type III domain-containing 5, PAI-1 plasminogen activator inhibitor-1, IL-1B Interleukin-1β, ENO2 enolase 2, PFKP platelet-type 6-phosphofructokinase, PFKFB4 6-phosphofructo-2-kinase/fructose-2,6-biphosphatase 4, and TBP TATA-binding protein.

| **Gene** | **Approach** | **∆c_t_** | **∆∆c_t_  (Calibrator1)** | **∆∆c_t_ (Calibrator2)** | **FC** | **± SD** | **ANOVA  *p*-Value** | |
| --- | --- | --- | --- | --- | --- | --- | --- | --- |
|  |  |  |  |  |  |  | ***** | ****** |
| **PFKP** | CN (calibrator1) | −3.116 | 0.000 |  | 1.000 | 0.285 | <0.001 |  |
|  | QN | −0.422 | 2.694 |  | 0.155 | 0.044 |  | <0.001 |
|  | CH (calibrator2) | −5.066 | −1.950 |  | 3.863 | 1.197 |  | <0.001 |
|  | QH | −1.865 | 1.251 |  | 0.420 | 0.141 |  | 0.006 |
|  |  | −1.865 |  | 3.201 | 0.109 | 0.039 |  | <0.001 |
| **PFKFB4** | CN (calibrator1) | 1637 | 0.000 |  | 1.000 | 0.301 | <0.001 |  |
|  | QN | 2283 | 0646 |  | 0.639 | 0.240 |  | 0.535 |
|  | CH (calibrator2) | −3034 | −4671 |  | 25.477 | 10.409 |  | <0.001 |
|  | QH | −1472 | −3108 |  | 8.624 | 3.860 |  | <0.001 |
|  |  | −1472 |  | 1563 | 0.338 | 0.170 |  | 0.017 |
| **ENO2** | CN (calibrator1) | 0.095 | 0.000 |  | 1.000 | 0.062 | <0.001 |  |
|  | QN | 0.195 | 0.100 |  | 0.933 | 0.167 |  | 1.000 |
|  | CH (calibrator2) | −3.001 | −3.096 |  | 8.551 | 1.749 |  | <0.001 |
|  | QH | −1.903 | −1.998 |  | 3.995 | 0.645 |  | <0.001 |
|  |  | −1.903 |  | 1.098 | 0.467 | 0.115 |  | 0.001 |
| **FNDC5** | CN (calibrator1) | 2.840 | 0.000 |  | 1.000 | 0.361 | 0.039 |  |
|  | QN | 2.396 | −0.444 |  | 1.361 | 0.420 |  | 0.857 |
|  | CH (calibrator2) | 2.692 | −0.148 |  | 1.108 | 0.323 |  | 1.000 |
|  | QH | 1.931 | −0.909 |  | 1.878 | 0.717 |  | 0.048 |
|  |  | 1.931 |  | −0.761 | 1.695 | 0.534 |  | 0.176 |
| **ANGPTL4** | CN (calibrator1) | −4.645 | 0.000 |  | 1.000 | 0.267 | <0.001 |  |
|  | QN | −2.705 | 1.940 |  | 0.261 | 0.066 |  | 0.001 |
|  | CH (calibrator2) | −5.810 | −1.165 |  | 2.242 | 0.700 |  | 0.057 |
|  | QH | −3.468 | 1.177 |  | 0.442 | 0.190 |  | 0.034 |
|  |  | −3.468 |  | 2.342 | 0.197 | 0.089 |  | <0.001 |
| **CFD** | CN (calibrator1) | 2.955 | 0.000 |  | 1.000 | 0.275 | 0.001 |  |
|  | QN | 4.486 | 1.531 |  | 0.346 | 0.101 |  | 0.008 |
|  | CH (calibrator2) | 2.390 | −0.565 |  | 1.479 | 0.421 |  | 1.000 |
|  | QH | 4.429 | 1.474 |  | 0.360 | 0.153 |  | 0.011 |
|  |  | 4.429 |  | 2.039 | 0.243 | 0.104 |  | 0.005 |
| **PAI−1** | CN (calibrator1) | −5.628 | 0.000 |  | 1.000 | 0.415 | 0.001 |  |
|  | QN | −4.234 | 1.394 |  | 0.380 | 0.140 |  | 0.012 |
|  | CH (calibrator2) | −5.908 | −0.279 |  | 1.214 | 0.405 |  | 1.000 |
|  | QH | −4.454 | 1.174 |  | 0.443 | 0.196 |  | 0.036 |
|  |  | −4.454 |  | 1.454 | 0.365 | 0.137 |  | 0.015 |
| **IL−1Β** | CN (calibrator1) | −3.217 | 0.000 |  | 1.000 | 0.243 | 0.039 |  |
|  | QN | −3.444 | −0.228 |  | 1.171 | 0.281 |  | 1.000 |
|  | CH (calibrator2) | −2.758 | 0.459 |  | 0.728 | 0.240 |  | 0.719 |
|  | QH | −2.690 | 0.526 |  | 0.694 | 0.193 |  | 0.365 |
|  |  | −2.690 |  | 0.067 | 0.954 | 0.336 |  | 1.000 |
